# In vitro and molecular docking studies of an anti-inflammatory scaffold with human peroxiredoxin 5 and tyrosine kinase receptor

**DOI:** 10.6026/97320630016929

**Published:** 2020-11-30

**Authors:** R Bharathi, N Santhi

**Affiliations:** 1 Research and development centre, Bharathiar university, Coimbatore, 641046, India; 2 Department Of Chemistry, Govt Arts College, C. Mutlur, Chidambaram, 608102, India

**Keywords:** in vitro anti-inflammatory, pyrazoline, docking, autodock

## Abstract

A new series of 4-(3-(2-amino-3,5-dibromophenyl)-1-(4-substitutedbenzoyl)-4,5-dihydro-1H-pyrazol-5-yl)benzonitrile (4a-h) compounds were synthesized and evaluated for in-vitro anti-inflammatory activities. The spectral (IR, NMR) and elemental analyses data of the product indicated the formation of new pyrazoles 4a-h. Compound 4e exhibited potent anti-inflammatory property with 85.45 % inhibitions. This value was compared with standard diclofenac sodium. This data is explained using molecular docking analysis of receptor- ligand binding. These results demonstrated that pyrazole derivatives are potential inhibitors of Human Peroxiredoxin 5 and Tyrosine kinase receptor in the treatment of inflammation related illness.

## Background

Inflammation is the natural defense mechanism of the body to deal with the infection and tissue damage [1]. However uncontrolled in diseases like chronic asthma, rheumatoid and osteo-arthritis, flammatory cascades is answerable for various multiple sclerosis, inflammatory bowel diseases and psoriasis, diabetic nephropathy [2] tumor initiation, and malignant progression [3]. Pain is the most common inflammatory indication needing medical attention and increase financial burden annually [4]. It is widely believed that deaths related to sepsis and sepsis will continue to rise. Research efforts in the field of sepsis have largely focused on the innate immune system and have conceptually viewed sepsis as a syndrome of hyper-inflammation [5,6]. Under this paradigm, overzealous activation of the host inflammatory response, ostensibly intended for pathogen eradication, becomes deregulated and consequently causes auto injury to the host which leads to multiple organ failure and death [7]. Peroxiredoxin 5 (PRDX5), also known as PrxV/ AOEB166/PMP20/ACR1, is a novel thioredoxin peroxidase widely expressed in mammalian tissues.z,4-6 PRDX5 may be addressed intracellularly to mitochondria, peroxisomes and the cytosol, suggesting that this peroxiredoxin may have an important role as antioxidant in organelles that are major sources of ROS, namely mitochondria and peroxisomes, and in the control of signal transduction due to its localization in the cytosol[8-10]. Moreover, the physiological importance of PRDX5 has recently been emphasized by its ability to prevent p53-induced apoptosis and to inhibit intracellular hydrogen peroxide accumulation by TNFα [11-12]. It is of interest to report the synthesis, biological evaluation and docking studies of a anti-inflammatory scaffold with binding features with protein target for further consideration.

## Materials and Methods:

Without pre-cleaning, all chemicals were purchased commercially. In the open capillary tube, melting points were identified and uncorrected. On PERKIN ELMER 240 CHN analyzer, elemental testing was carried out. On a Shimadzu FTIR spectrophotometer in the 400-4000 cm-1 range with KBr pellets, an FT-IR spectrum of title compounds was recorded. The 1H NMR spectrometer was recorded with the solvents of DMSO and CDC13 on a 400 MHz NMR BRUKER AVANCE. The ppm was reported to have chemical shifts. As an internal reference to every NMR spectrum, tetramethylsilane (TMS) was used, with chemical shifts reported as standard in the case of units (parts per million).

## Synthesis of 4-(3-(2-amino-3,5-dibromophenyl)-1-(4-substitutedbenzoyl)-4,5-dihydro-1H-pyrazol-5-yl)benzonitrile:

Chalcones (3) were obtained by condensation in the first step 2-amino-3, 5-dibromobenzaldehyde (2) 4-acetylbenzonitrile (1). Then, a mixture in 25 mL of acetic acid was refluxed with 3 (0.01 mol) of aryl hydrazide (0.01 mol), 8 hours. Filtration and purification of the precipitate were performed by ethanol recrystallation. Compound synthetic pathway is shown in Scheme 1 4a-h.

## Analysis of the anti-inflammatory activities using HRBC membrane stabilization Method:

The antiinflmmatory activity of compounds 4a-h was assessed by in vitro HRBC membrane stabilization method. Blood was collected from healthy volunteers. Th collected blood was mixed with equal volume of Alsever solution (dextrose 2%, sodium citrate 0.8%, citric acid 0.05%, sodium chloride 0.42%, and distilled water 100 mL) and centrifuged with isosaline. To 1 mL of HRBC suspension, equal volume of test drug in three diffrent concentrations, 100, 250, and 500° µg/mL, was added. All the assay mixtures were incubated at 37°C for 30 minutes and centrifuged. The haemoglobin content in the supernatant solution was estimated by using spectrophotometer at 560 nm [22]. Here, the negative control used was Alsever's solution with blood in it and it contained no Aspirin [15].

## Molecular docking:

Crystal structures of the protein complex used in this study were obtained from the the protein data bank (www.rcsb.org/pdb)[16]. Docking calculation was carried out using autodock 4.2 [17, 18]. Gasteiger partial charges were added to the ligand atoms. Non­polar hydrogen atoms were merged, and rotatable bonds were defined. Docking calculations were carried out on DNA gyrase protein model. Essential hydrogen atoms, Kollman united atom type charges, and solvation parameters were added with the aid of auto-Dock tools [16]. Affinity (grid) maps of xx Å grid points and 0.375Å spacing were generated using the autogrid program [16]. Auto-Dock parameter set­ and distance­dependent dielectric functions were used in the calculation of the vander Waals and the electrostatic terms, respectively. Docking simulations were performed using the Lamarckian genetic algorithm (LGA) and the Solis & Wets local search method [19]. Initial position, orientation, and torsions of the ligand molecules were set randomly. All rotatable torsions were released during docking. Each docking experiment was derived from 2 different runs that were set to terminate after a maximum of 250000 energy evaluations. The population size was set to 1 50. During the search, a translational step of 0.2 Å, and quaternion and torsion steps of 5 were applied.

## Results and Discussion:

**Figure 1:**
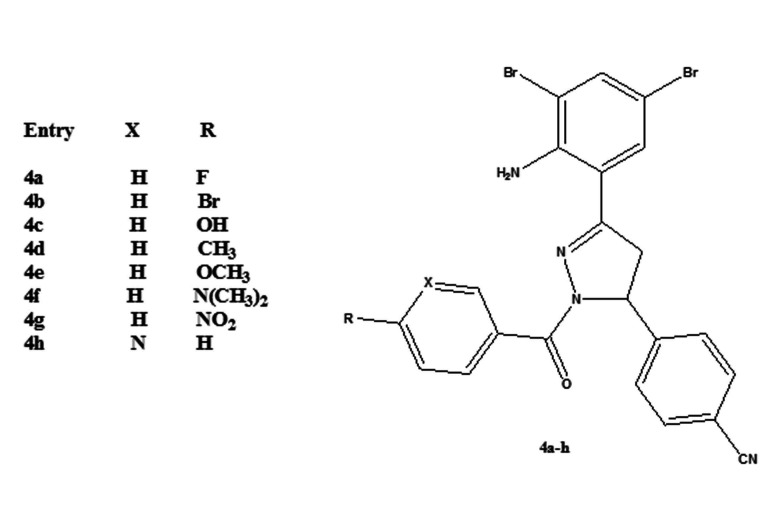
Molecular structure of 1,3,5-trisubstituted pyrazoline derivatives 4a-h

Our research work started with the preparation of 4-(3-(2-amino-3, 5-dibromophenyl)-1-(4-substitutedbenzoyl)-4,5-dihydro-1H-pyrazol-5-yl) benzonitrile (4a-h). Pyrazole analogues (4a h) were synthesized by condensation of (E)-4-(3-(2-amino-3,5 dibromophenyl)acryloyl) benzonitrile and aryl hydrazide in the presence of acetic acid using conventional heating (Figure 1). The spectral characterization (IR, NMR) and elemental analysis data of the product indicated the formation of new pyrazoles 4a-h. The absorption bands in the region 1598-1620 cm-1 for C=N stretching group [20] in pyrazoles 4a-h. A strong absorption bands in the region 2351-2360 cm-1 are ascribed to CN stretching. The carbonyl stretching vibrations are observed in the around1660 cm-1. In title compounds, N-H [21,22] stretching vibration was observed ca. 3456 cm-1, which supports the formation of pyrazole ring. The 1H NMR spectra revealed the presence of a two doublet of doublet signals around 3.74 and 3.09 ppm are easily assigned to proton CHB and CHA protons, respectively in pyrazole molecule. The benzylic proton in pyrazole moiety appeared as a doublet of doublet in the region 4.86-3.95 ppm. The -NH2 protons are noticed by proton NMR spectra as a sharp singlet around 5.42-5.90 ppm. Additionally, aromatic protons are resonating as multiplets in the range between 6.90-8.69 ppm. These signals confirm the formation of pyrazoles 4a-h.

**Figure 2:**
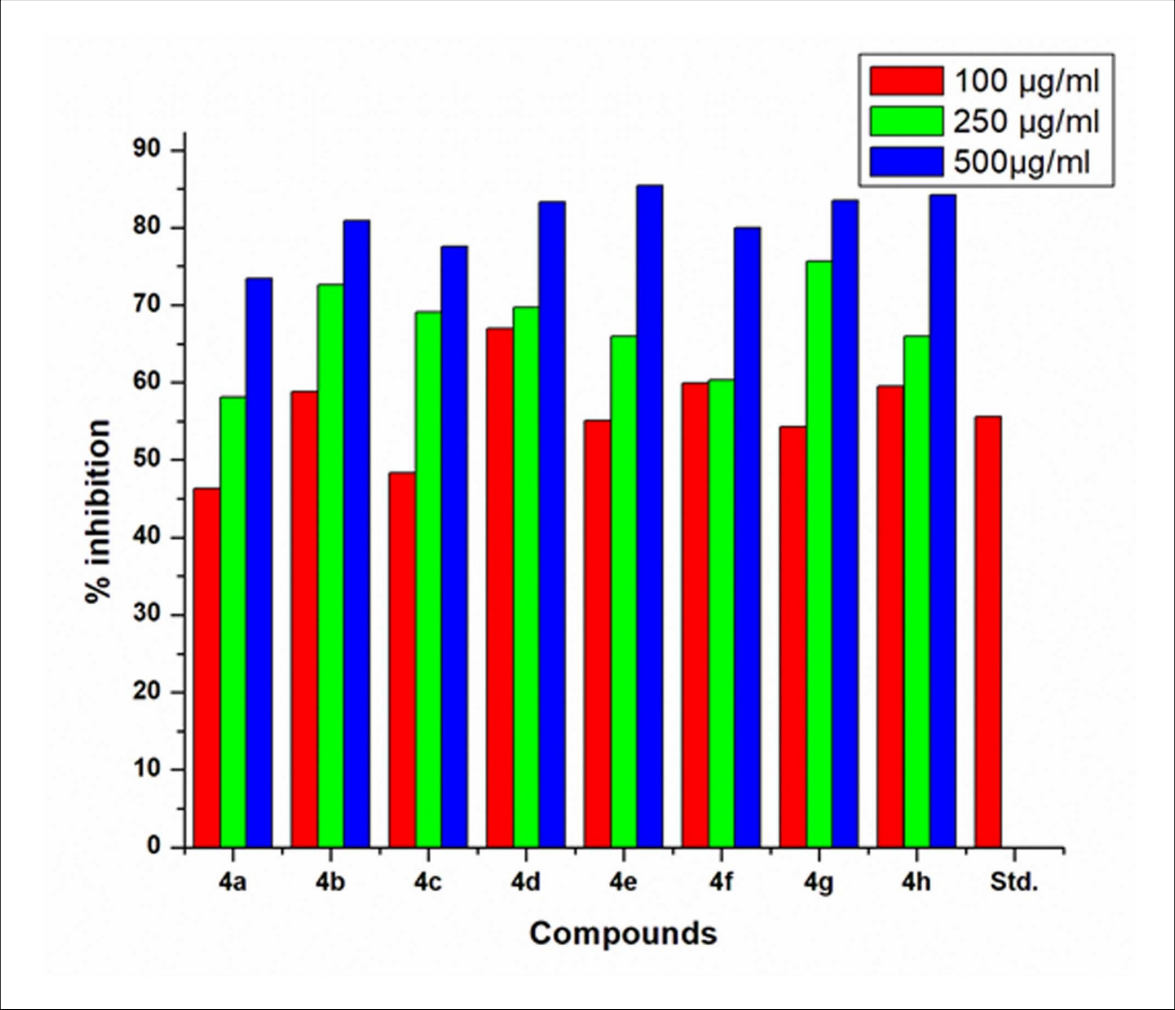
Antioxidant inhibition profile of tested compounds at 100,250 and 500 µg/ml concentrations

The designed compounds were studied for in vitro anti-inflammatory activity by HRBC membrane stabilization method. The anti-inflammatory activity data (Table 1 - see PDF) indicated that all the test compounds exhibited significant activity when compared to standard diclofenac sodium. The data obtained were presented in Table 1 (see PDF). All tested compounds offered adequate protection in a dose-dependent manner. The activity was increased with increasing concentration. The result of the in vitro membrane stabilization activity of synthesized pyrazoline (4a-h) is presented in Table 1(see PDF) and Figure 2. According to these results all the compounds showed dose dependent inhibition of hemolysis. Compound 4f (IC50 = 159.1µg/ml) and 4b (IC50 = 180.3 µg/ml) displayed very good activity among the series as compared to standard Diclofenac sodium (IC50 = 127.3 µg/ml). Other compound 4g (IC50 = 185.0µg/ml) and 4d (IC50 = 192µg/ml) showed moderate activity and 4c (IC50 = 352.1µg/ml) and 4h (IC50 = 370.6µg/ml) had exhibited lower anti-inflammatory activity as compared to standard DCS.

**Figure 3:**
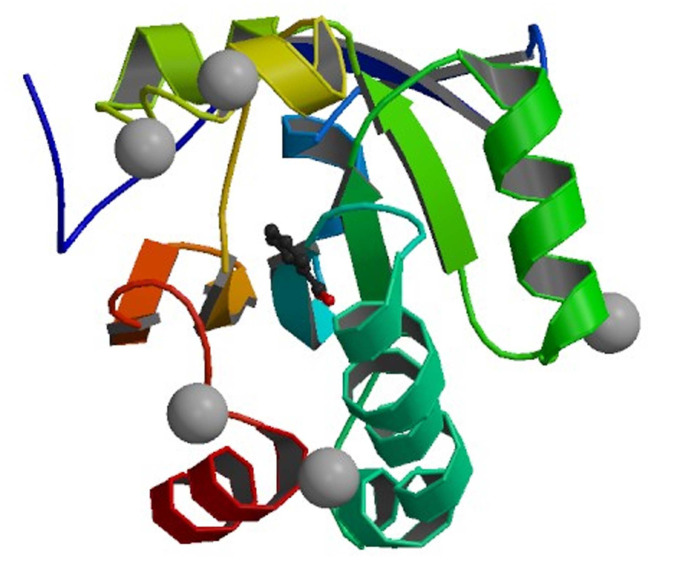
3D view of human peroxiredoxin 5 receptor (PDB ID: 1HD2)

**Figure 4:**
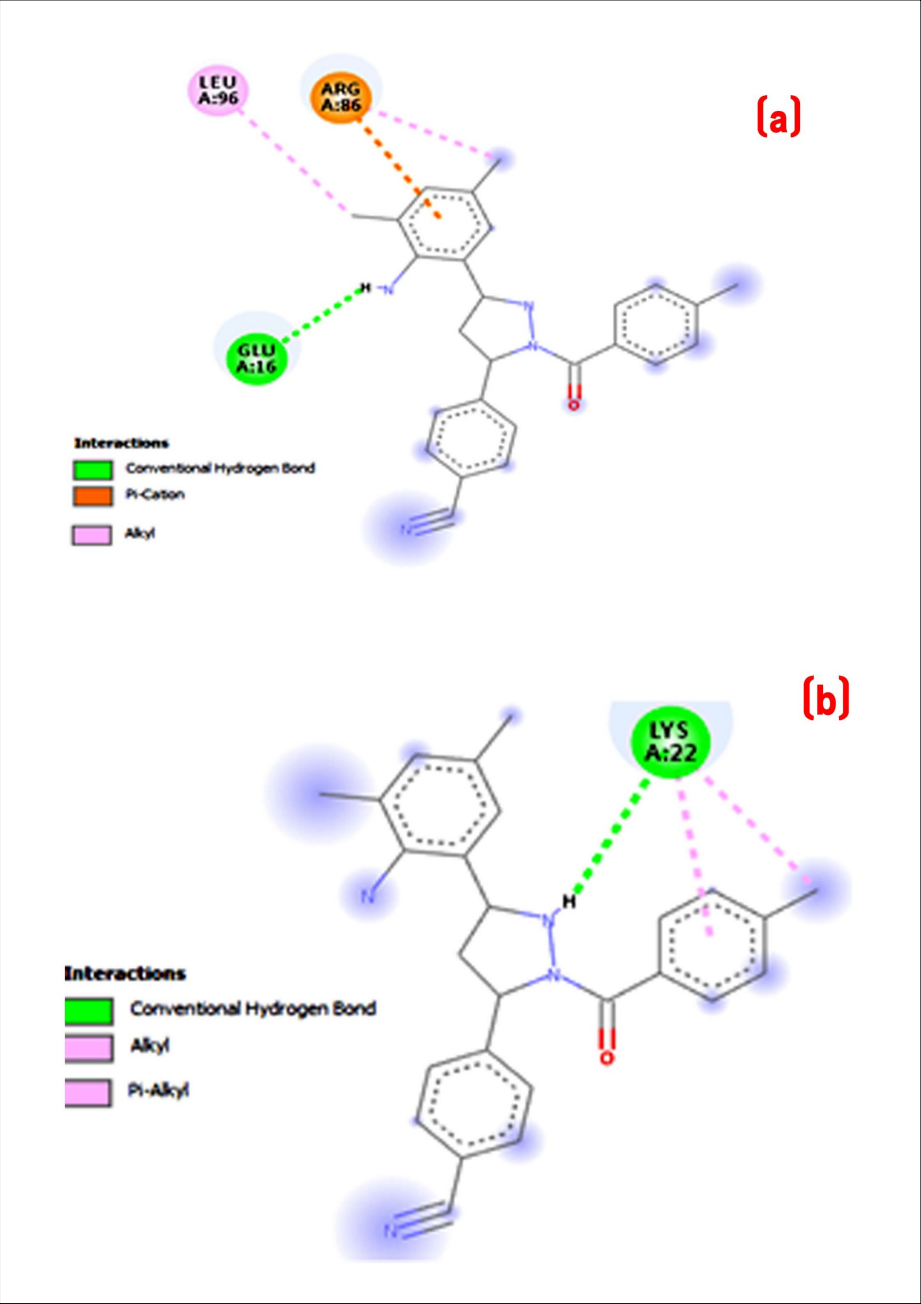
2D Docked conformation of most active compound 4d with protein (a) human peroxiredoxin 5 receptor; (b) tyrosine kinase HCK receptor

**Figure 5:**
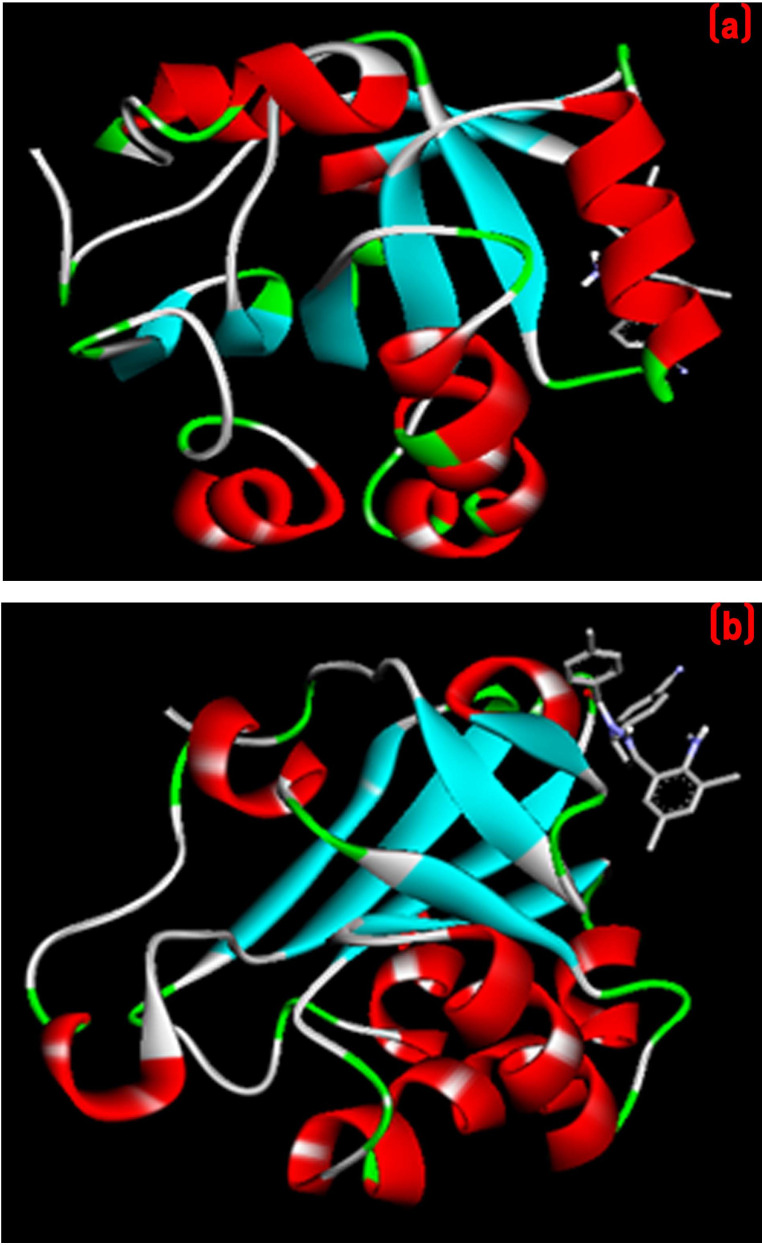
Ribbon binding model compound 4d (a) human peroxiredoxin 5 receptor (b) tyrosine kinase HCK receptor

In an attempt to explain how our designed compounds interact with active site of the Human Peroxiredoxin 5 (Figure 3) flexible docking simulations were carried out to predict the receptor-ligand interactions by using autodock 4.2. The protein crystal structure Human Peroxiredoxin 5 (PDB ID: 1HD2) was obtained from the PDB (www.rcsb.org/pdb). The graphical depiction of protein is mentioned in Figure 2. Compounds 4a-h have scores ranged from - 5.01 to -6.06 Kca/mol. According to the docked structure of compound 4a forms one hydrogen bond with THR147. Further interactions, including hydophobic and Alkyl/Pi-Alkyl were observed giving a total binding energy value of -5.01 kcal/mol with protein. Replacing of fluoro group by bromo (compound 4b) leads to increasing binding energy (-5.60 kcal/mol). Compound 4b also showed hydophobic interactions with PHE128, ILE119, PRO45 and LEU116 amino acids. The hydroxyl substituted compound 4c showed binding energy value of -5.95 kcal/mol and makes one hydrogen bond with THR147. It was noticeable that compounds with methyl group substitution (4d) at phenyl group had a significant impact on the activity. Compound 4e has highest binding scores in this experiment as shown Table 2 (see PDF). Figure 4a and Figure 5a shows three-dimensional binding pose of two active compound 4d with Human Peroxiredoxin 5. A hydrophobic interaction was observed between the ligand and protein. Compound 4e also showed π-alikyl interactions with THR44 and PRO40 amino acids. The compounds 4f and 4g showed similar binding energy. The replacing of phenyl group by pyridine ring (compound 4h) showed binding energy about -5.61 kcal/mol.

**Figure 6:**
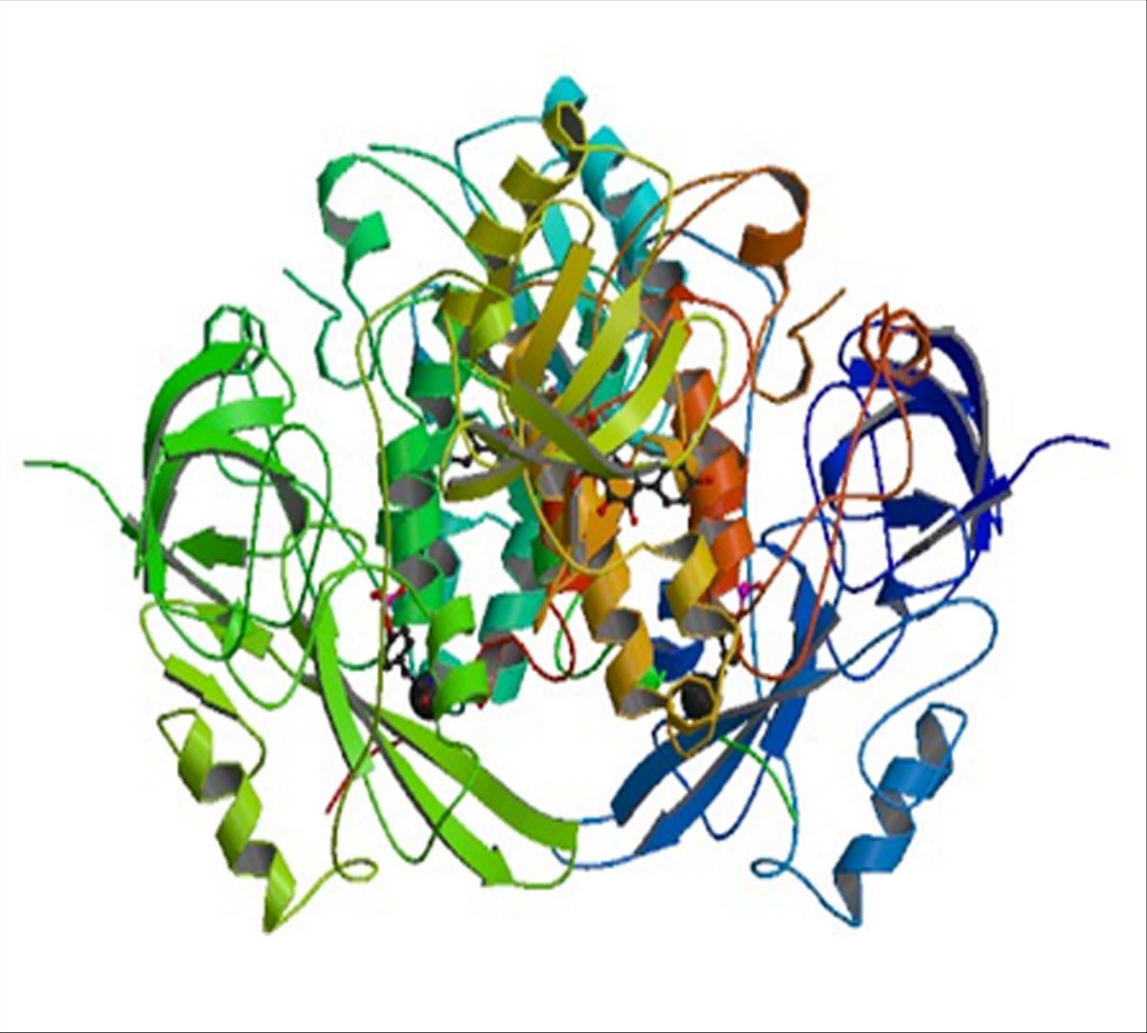
3D view of tyrosine kinase HCK receptor (PDB ID: 2HCK)

Figure 4b and Figure 5b shows three-dimensional binding pose of two active compound 4d with tyrosine kinase HCK. The X-ray crystal structure of protein tyrosine kinase Hck (PDB: 2HCK) is shown in Figure 6. The newly designed compounds 4a-h was docked into tyrosine kinase Hck protein to understand the binding interactions. Compound 4a has binding energy -5.41 kcal/mol with three hydrogen bonds viz. THR179, GLN529 and GLN528, when introducing bromo substitution (4b) in phenyl group. It is pertinent to note that the ligand 4b exhibit nice binding energy -5.84 kcal/mol.

As depicted in Table 3 compound 4b showed hydrogen interactions with GLN528, GLN526 and GLN529. In addition it has π-alkyl interaction with LYS203, THR523 residues. The compound 4c show nice binding energy -6.63 kcal/mol and makes hydrogen bond with GLN526 andGLN525 amino acid residues. Further, it makes π-alkyl interaction with GLU524, THR523, LYS203, THR179 and ARG155 residues. The binding pattern of ligand 4d with protein clearly revealed that the ligand polar interactions with ARG205, ARG175 and SER185. In addition, it showed π-alkyl interaction with LYS203 and THR523. As seen from Table 3 (see PDF), ligand 4e showed higher (-7.4 kcal/mol) binding affinity within the protein and it forms hydrogen bond interaction with THR179 amnio acid. A polar interaction was observed between the ligand and protein. Compound 4e also showed π-alikyl interactions with GLN529, HIS201, THR523, ARG155 and ARG175 amino acids. The compounds 4f and 4g showed binding energies -5.99 and -5.23 kcal/mol. In this series, compound 4g shows the least binding energy. The pyridine substituted compound 4h gives binding energy -5.94 kcal/mol and makes one hydrogen bond with GLN526 amino acid residue. Further, it makes π-alkyl interaction with ARG155, ARG205, ARG175, SER185 and GLN529 residues.

## Conclusion

We describe the synthesis, biological evaluation (85.45% inhibition) and docking studies of a anti-inflammatory scaffold [74-(3-(2-amino-3, 5-dibromophenyl)-1-(4-substitutedbenzoyl)-4,5-dihydro-1H-pyrazol-5-yl) benzonitrile] with molecular binding features with the protein targets such as the human peroxiredoxin 5 and tyrosine kinase Hck for further consideration.
